# Effect on OPU/IVEP success of different applications for synchronizing follicular waves prior to superstimulation in holstein heifers

**DOI:** 10.5194/aab-68-101-2025

**Published:** 2025-02-11

**Authors:** Muhammed Furkan Çiftçi, Ömer Faruk Yeşilkaya, Sakine Ülküm Çizmeci, Maide Gölbaşı, Ayşe Sarı, Dursun Ali Dinç

**Affiliations:** 1 Department of Obstetrics and Gynecology, Faculty of Veterinary Medicine, Selçuk University, Konya, Türkiye; 2 Department of Obstetrics and Gynecology, Faculty of Veterinary Medicine, Muğla Sıtkı Kocman University, Muğla, Türkiye; 3 Department of Reproduction and Artificial Insemination, Faculty of Veterinary Medicine, Necmettin Erbakan University, Konya, Türkiye

## Abstract

The aim of this study is to determine the effects of different treatments used to synchronize follicular waves prior to superstimulation of responses and in vitro embryo production (IVEP). The study used 12 healthy Holstein heifers. In GnRH + P4, an intravaginal progesterone (P4) device was inserted into the vagina on a random day of the estrous cycle, and 10 µ g of the gonadotropin-releasing hormone (GnRH) was administered intramuscularly at the same time. After these applications, 25 mg of PGF2 α was administered intramuscularly on the third and fourth days of treatment. The intravaginal progesterone device was removed on day 5. At the same time, 125 µ g of follicle-stimulating hormone (FSH) was administered intramuscularly as a single dose. Two days after the FSH injection, oocytes were retrieved using transvaginal ultrasound. In contrast to GnRH + P4, hCG + P4 was performed with 1500 IU hCG (human chorionic gonadotropin) injection instead of GnRH. In DFR + P4, dominant follicles were removed. In the control group, the oocytes were collected on a random day of the estrous cycle. The ovarian follicles were measured by ultrasound prior to oocyte collection. The number of medium-diameter follicles (3–8 mm) was found to be greater in hCG + P4 and DFR + P4 than in the control group ( p<0.05 ). It was determined that the numbers of viable oocytes, Grade A oocytes, cleaved oocytes, and blastocysts obtained per ovum pickup (OPU) were greater in hCG + P4 and DFR + P4 than in the control group ( p<0.05 ). In addition, the numbers of Grade A oocytes and blastocysts obtained per OPU were greater in the DFR + P4 group than in the GnRH + P4 group ( p<0.05 ). As a result, it was thought that synchronization of follicular wave emergence with DFR and hCG in the short synchronization protocols used before superstimulation could increase the success rate of OPU/IVEP programs.

## 1 Introduction

In vitro embryo production (IVEP) allows the number of high-yielding animals to be increased rapidly. Therefore, in order to increase the reproductive efficiency of cattle, biotechnological methods are of great importance (Monteiro et al., 2017). The transvaginal follicle aspiration technique (ovum pickup – OPU) is widely used to obtain oocytes from high-yielding animals for in vitro embryo production. As the OPU/IVEP technique can produce a much larger number of embryos than the conventional method, a rapid increase in in vitro embryo production has recently been observed, according to data from the International Embryo Technologies Society (IETS) (Santl et al., 1998; Viana, 2023).

Follicular wave studies have stated that the dominant follicle causes the other follicles to regress and suppresses the appearance of the next follicular wave. It has been reported that higher oocyte retrieval rates and higher-quality oocytes are obtained due to the absence of atretic events in aspirations performed during the follicular growth phase when there is no dominant follicle (Bacelar et al., 2010; Gimenes et al., 2015). For this reason, hormonal treatments are frequently used before OPU applications, and the aim is to increase the number of follicles suitable for aspiration during oocyte retrieval. The majority of oocytes obtained in IVEP are collected prior to ovulation from follicles 3 to 8 mm in diameter (Dieleman et al., 2002). The superstimulation response is negatively affected by the presence of a dominant follicle in the ovary during superstimulation. Therefore, superstimulation before OPU is used in conjunction with synchronization protocols. The effects of the dominant follicle can be eliminated by hormone stimulation or dominant follicle removal (DFR) (Ginther et al., 1989; Ko et al., 1991; Adams, 1994).

The OPU technique allows oocytes to be collected from the same animal at short intervals (Boni, 2012). Short-term hormone treatments are being developed so that more OPU sessions can be applied to high-yielding animals. The best time to start superstimulation is when a new follicular wave emerges (Nasser et al., 1993). Methods such as the gonadotropin-releasing hormone (GnRH), dominant follicle removal, and a combination of estradiol (EB) and progesterone are used to induce a new follicular wave. It is reported that the combination of estradiol and progesterone is the most successful hormonal treatment for follicular wave synchronization (Nasser et al., 1993; Bergfelt et al., 1994; Bó et al., 1996, 2002; Boni, 2012). Accordingly, estradiol is used in many of the short synchronization protocols followed before superstimulation (Vieira et al., 2014; De Carvalho et al., 2019). However, the European Union has banned the use of estradiol in animals for food safety reasons. Alternative synchronization protocols prior to superstimulation in OPU/IVEP programs are therefore required (Bó and Menchaca, 2023; Ciftci and Dinc, 2023; Huang et al., 2023).

The aim of this study was to determine the effect of different treatments (GnRH, hCG (human chorionic gonadotropin), and DFR) used to synchronize the follicular wave emergence prior to superstimulation on the superstimulation response and success of in vitro embryo production.

## 2 Materials and methods

This study was approved by the Selçuk University Local Ethics Committee for Animal Experiments (approval no. 2024/01/17).

### 2.1 Experimental animals

The study was conducted on a genomic selection farm in Türkiye. In this study, 12 Holstein heifers without health problems were used. The body condition scores of the heifers ranged from 2.5 to 3.5. In order to prevent individual differences in oocyte yield from influencing the results of the study, the same animals were rested and four sessions were convened. There were 21 d between the sessions. The heifers were assigned to a four-treatment crossover design study. The study was conducted in four separate cohorts (three heifers per cohort). All the heifers received all the treatments. The animals were fed ad libitum. The ration included alfalfa silage, corn silage, hay, alfalfa, concentrated feed, and vitamin and mineral supplements.

### 2.2 Experimental design and treatment protocols

GnRH + P4 ( n=12 ): on a random day of the estrous cycle, an intravaginal progesterone device (containing 1.38 g progesterone; ClDR 1380^®^, Zoetis, New Zealand) was inserted into the vagina, and 10 µ g of GnRH (buserelin acetate, Receptal^®^, Intervet, Germany) was administered intramuscularly at the same time. After these applications, 25 mg of PGF2 α (Dinoprost, Dinolytic^®^, Zoetis, USA) was administered intramuscularly on days 3 and 4. The intravaginal progesterone device was removed on day 5. At the same time, a single dose of 125 µ g of follicle-stimulating hormone (FSH) (Stimufol^®^, Belgium) was administered intramuscularly. Oocytes were collected 2 d after FSH injection.

hCG + P4 ( n=12 ): in contrast to the protocol described for the GnRH + P4 group, 1500 IU hCG (Chorulon^®^, MSD Animal Health, Germany) was administered intramuscularly on a random day of the estrous cycle.

DFR + P4 ( n=12 ): dominant follicles were removed on a random day of the estrous cycle (DFR: all follicles over 8 mm were removed; De Roover et al., 2005). The same protocol as described for the GnRH + P4 group was then followed before oocyte retrieval.

Control ( n=12 ): on a random day of the estrous cycle, oocytes were collected from the animals without any pretreatment.

**Figure 1:**
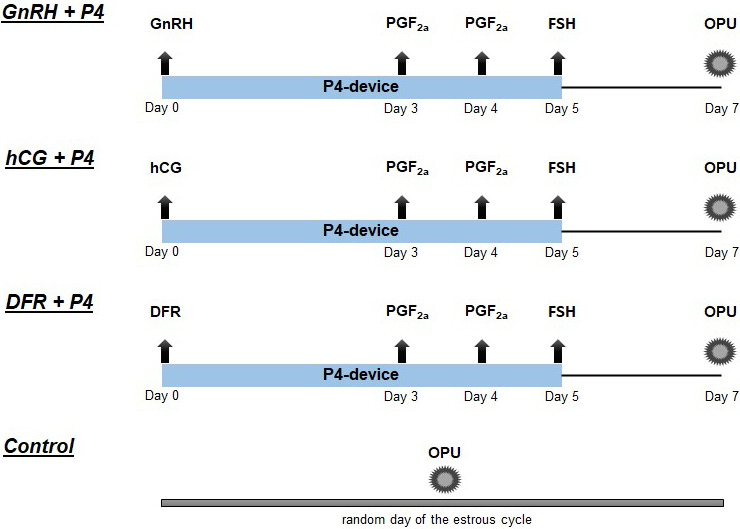
The experimental groups and synchronization superstimulation protocols for OPU/IVEP (DFR: dominant follicle removal; hCG: human chorionic gonadotropin; P4: progesterone device; PGF2 α : prostaglandin F2a; GnRH: gonadotropin-releasing hormone; FSH: follicle-stimulating hormone).

The experimental groups and synchronization superstimulation protocols for the OPU/IVEP program are schematized in Fig. 1.

### 2.3 Ovum pickup

For transvaginal oocyte retrieval, a combination of a catheter-aspiration device (aspiration pump for bovine OPU, 230 V, Minitube), a real-time ultrasound device (Esaote MyLab TwiceVet, 5001), and a 4.0–9.0 MHz micro-convex probe (SC3123 VET, Esaote) was used. Before oocyte retrieval, follicles in the ovary were classified according to their diameter as small (0–3 mm), medium (3–8 mm), or large ( < 8 mm). All follicles larger than 2 mm in the ovary that could be aspirated were aspirated using a catheter with a 20-gauge needle and a special micro-convex vaginal probe.

### 2.4 Morphological classification of oocytes

Cumulus–oocyte complexes (COCs) were examined under a stereomicroscope (S ApoE, Leica). The oocytes obtained were classified according to their morphological characteristics. Morphological characteristics such as cumulus cell compactness, cumulus cell layer, and cytoplasmic homogeneity were evaluated in the collected oocytes. The quality of the oocytes was classified as very good (Grade A), good (Grade B), moderate (Grade C), or poor (Grade D) (Gordon, 2003; Petyim et al., 2003). Grade A, B, and C oocytes were evaluated as viable for in vitro embryo production.

### 2.5 In vitro embryo development

In this study, embryo production was performed using media (BO-IVM, BO-IVF, BO-IVC, BO-Wash, BO-Oil, and BO-SemenPrep) produced by a commercial company (IVF Bioscience). Firstly, COCs, whose quality was assessed after OPU, were washed three times in the oocyte washing medium (BO-Wash). The COCs were then placed in an in vitro maturation medium (BO-IVM) and incubated at 38.5 °C and 5.5 % CO_2_ for 20–22 h. Following in vitro maturation, the oocytes were transferred to the in vitro fertilization medium and kept in the incubator until the semen was prepared. Semen from the same sire was used in the in vitro fertilization phase throughout the study. The commercial company's medium (BO-SemenPrep) was used for the semen preparation. A sperm-washing procedure was applied. Afterwards, the number of spermatozoa was calculated to be 1 million per milliliter and was added to the in vitro fertilization medium containing cumulus–oocyte complexes (incubated for 20 h at 38.5 °C and 5 % CO_2_). Oocytes were vortexed to remove cumulus cells. Possible zygotes were transferred to the in vitro culture medium (BO-IVC) coated with mineral oil (BO-Oil). Possible zygotes in the in vitro culture medium (BO-IVC) were incubated at 38.5 °C, 6 % CO_2_, and 6 % O_2_ for 7–9 d. The quality and developmental stages of the embryos were evaluated according to the IETS (Bó and Mapletoft, 2013; Alkan et al., 2023).

**Table 1:** Follicle numbers during OPU of the groups (mean ± standard deviation).

	0–3 mm	3–8 mm	> 8 mm	Total
GnRH + P4	4.75 ± 1.81^a^	14.25 ± 6.39^ab^	0.67 ± 0.65^ab^	19.66 ± 6.94
hCG + P4	4.58 ± 2.57^a^	16.67 ± 6.78^a^	0.16 ± 0.38^a^	21.41 ± 8.63
DFR + P4	6.00 ± 1.95^a^	17.83 ± 8.45^a^	0.25 ± 0.45^a^	24.08 ± 8.50
Control	9.75 ± 7.78^b^	9.75 ± 3.10^b^	1.33 ± 1.55^b^	21.43 ± 8.17

^a−c^ Statistical differences between the columns ( p<0.05 ).

### 2.6 Statistical analysis

SPSS 25.0 (IBM Corp., 2017, IBM SPSS Statistics for Windows, version 25.0; Armonk, NY, USA) was used for the statistical analysis of the data. A Shapiro–Wilk test was used to check the homogeneity of the variances and the normality assumptions of the variables. Variables that did not have a normal distribution were presented as the median (minimum or maximum), and a Kruskal–Wallis test was used for the analysis of variance. Normally distributed variables were presented as the mean ± standard deviation (SD), and ANOVA with a post hoc Tukey test was used to evaluate the variables. Proportions were analyzed using a Chi-squared test. The significance level of the tests was accepted as p<0.05 .

## 3 Results

There were similar results between the groups for the total number of antral follicles at the time of oocyte retrieval. The number of large-sized follicles ( > 8 mm) was found to be greater in the control group than in the hCG + P4 and DFR + P4 groups ( p<0.05 ). It was determined that the number of medium-sized follicles (3–8 mm) was greater in the hCG + P4 and DFR + P4 groups than in the control group. In addition, there was no statistical difference in the number of medium-sized follicles of the GnRH + P4 group and the control group. The number of small-sized follicles (0–3 mm) was found to be greater in the control group than in all the other groups ( p<0.05 ). The number of follicles is shown in Table 1 according to their diameters in the ultrasound examination performed before follicular aspiration.

**Table 2:** The distribution of oocytes in the groups, presented based on their quality (median – minimum or maximum).

	Grade A	Grade B	Grade C	Grade D	Total
GnRH + P4	2 (0–7)^ab^	2 (0–5)	3 (1–16)	3 (0–16)	11 (2–28)^ab^
hCG + P4	3 (1–18)^bc^	2 (0–6)	4 (0–9)	4 (1–13)	12 (4–40)^b^
DFR + P4	5 (1–11)^c^	2 (1–10)	3 (0–7)	4 (1–11)	12 (7–33)^b^
Control	1 (3–0)^a^	3 (0–7)	2 (0–7)	2 (2–9)	7 (3–19)^a^

^a−c^ The statistical differences between the columns ( p<0.05 ).

The number of Grade A oocytes obtained was found to be lower in the control group than in the hCG + P4 and DFR + P4 groups ( p<0.05 ). In addition, it was found that the number of Grade A oocytes obtained in the DFR + P4 group was greater than that obtained in the GnRH + P4 group ( p<0.05 ). There was no statistical difference between the groups in terms of the number of Grade B, C, and D oocytes retrieved. The total number of oocytes was found to be greater in the hCG + P4 and DFR + P4 groups than in the control group. The total number of oocytes was found to be similar in the GnRH + P4 and control groups. The number and quality of the oocytes retrieved after OPU application are shown in Table 2.

**Table 3:** OPU/IVEP results in the groups after the applications (mean ± standard deviation).

	GnRH + P4	hCG + P4	DFR + P4	Control
Total oocytes	147	192	190	115
Viable oocytes	97	134	130	79
Viable oocytes/OPU	8.08 ± 4.85^ab^	11.16 ± 9.34^b^	10.83 ± 6.89^b^	6.58 ± 3.36^a^
Cleaved oocytes/OPU	5.16 ± 3.56^ab^	8.08 ± 6.61^b^	8.16 ± 5.16^b^	4.08 ± 2.19^a^
Cleavage rate (%)	63.91^a^	73.13^b^	75.38^b^	62.02^a^
Blastocysts/OPU	1.91 ± 1.08^ab^	3.00 ± 2.21^bc^	3.33 ± 1.49^c^	1.58 ± 0.90^a^
Blastocyst rate (%)	23.71^a^	26.86^ab^	30.76^b^	24.05^ab^

^a−c^ Statistical differences between the rows ( p<0.05 ).

The count of viable oocytes was greater in the hCG + P4 and DFR + P4 groups than in the control group ( p<0.05 ). Similar results were obtained in terms of the number of viable oocytes in the groups where follicular wave synchronization and superstimulation (GnRH + P4, hCG + P4, and DFR + P4) were applied. There was no statistical difference between the synchronization and superstimulation groups in terms of the number of cleaved oocytes per OPU. However, it was determined that the number of cleaved oocytes in the hCG + P4 and DFR + P4 groups was greater than in the control group ( p<0.05 ). The cleavage rate was found to be higher in the hCG + P4 and DFR + P4 groups than in the control and GnRH + P4 groups ( p<0.05 ). The number of blastocysts per OPU was found to be greater in the DFR + P4 group than in the GnRH + P4 and control groups ( p<0.05 ). It was found that the number of blastocysts per OPU was lower in the control group than in the hCG + P4 group ( p<0.05 ). However, the number of blastocysts per OPU was not statistically different in the GnRH + P4 and hCG + P4 groups. The blastocyst rate was higher in the DFR + P4 group than in the control group ( p<0.05 ). The OPU/IVEP results in the groups are shown in Table 3.

## 4 Discussion

This study evaluated the effects of different methods used to synchronize follicular waves prior to superstimulation in OPU applications on superstimulation response, oocyte yield, and in vitro embryo production.

The antral follicle count (AFC) is one important factor used to evaluate donor animals for oocyte yield. In general, genetic and environmental factors influence the AFC in the ovaries of donor animals. Pre-OPU treatments are used to increase the number of follicles with suitable diameters in the ovary (De Roover et al., 2005; Garcia et al., 2020). Follicle diameter is an important parameter for the developmental competence of oocytes used for in vitro embryo production. It is stated that oocytes obtained from follicles smaller than 3 mm in bovine ovaries have a low developmental capacity. It is reported that oocytes obtained from medium-diameter follicles (5–10 mm) have a higher rate of reaching the blastocyst. For this reason, synchronization and superstimulation applications before OPU focus on increasing the number of medium-diameter follicles (3–8 mm) (Dieleman et al., 2002; Sirard, 2012). Da Silva et al. (2017) found in their study that synchronization and superstimulation applications did not affect the total number of follicles but increased the proportion of medium-sized follicles. In a separate study, it was demonstrated that the administration of repeated doses of FSH led to a notable increase in the number of medium-sized follicles (Nawaz et al., 2021). Vieira et al. (2014) also observed that synchronization of estradiol and progesterone prior to superstimulation increased the number of medium-sized follicles (6–10 mm). In another study that investigated the effects of dominant follicle removal before superstimulation in OPU applications, it was observed that synchronization application resulted in an increase in the number of medium-sized follicles (6–10 mm) (Hayden et al., 2022). In this study, the groups showed similar results in terms of the total number of follicles during oocyte retrieval. During OPU, the hCG + P4 and DFR + P4 groups had a greater number of medium-sized follicles (3–8 mm) compared to the control group. Therefore, using hCG and DFR applications for follicular wave synchronization is considered effective in stimulating the formation of new follicular waves.

Synchronization prior to superstimulation prevents the formation of atretic and dominant follicles by creating a homogeneous follicle population and initiating a new follicular wave. Follicular wave synchronization is used to maximize the superstimulation response and increase the number of antral follicles present at the beginning of the stimulation. The quality and recovery rate of oocytes in OPU/IVEP programs are affected by the phase of the follicular wave during the application (Seneda et al., 2001; Aerts and Bols, 2010). A study on beef cattle reported that the quality of COCs increased with follicular wave synchronization (EB + P4) before OPU (Ongaratto et al., 2018). Another study was conducted to investigate the effect of synchronization (DFR) before superstimulation in OPU application. The results showed that synchronization application increased the number of viable oocytes (Hayden et al., 2022). Reis et al. (2010) reported that follicular wave synchronization increased oocyte yield in Brangus breed donors. Their study found that the groups synchronized with hCG + P4 and DFR + P4 had a greater number of Grade A oocytes, total oocytes, and viable oocytes compared to the control group. Additionally, it was determined that the number of Grade A oocytes in the DFR + P4 group was greater than in the GnRH + P4 group. It is thought that stimulating the formation of a new follicular wave before superstimulation may increase oocyte yield by reducing the number of atretic follicles.

The follicular phase in the donor's ovary during oocyte collection affects the oocyte quality in OPU/IVEP applications. Improving the oocyte quality and reducing the rate of atretic follicles through pre-OPU application enhances the success of in vitro embryo production (Hendriksen et al., 2000). Cavalieri et al. (2018) assert that follicular wave synchronization (EB + P4) before OPU increases the rate of reaching a blastocyst. In a similar study, it was reported that follicular wave synchronization before OPU increased the blastocyst yield (Cavalieri et al., 2018). In a study where the dominant follicle was removed prior to FSH in OPU/IVEP, the embryo yield was found to increase significantly (De Ruigh et al., 1996). This study found that the cleavage rate and the number of blastocysts obtained per OPU were higher in the hCG + P4 and DFR + P4 groups compared to the control group. Furthermore, it was determined that the number of blastocysts obtained per OPU in the DFR + P4 group was greater than that of the GnRH + P4 group. This study supports previous research indicating that synchronizing pre-OPU follicular waves can increase oocyte yield and improve the success of in vitro embryo production.

## 5 Conclusions

Hormonal treatments and DFR had no effect on the AFC at the time of oocyte retrieval. However, the use of DFR and hCG applications to induce follicular wave emergence before superstimulation resulted in an increase in the number of follicles with the ideal diameter for OPU. It is thought that superstimulation applications performed as a result of stimulating follicular wave formation with DFR and hCG will increase oocyte yield and IVEP success. As a result, it has been concluded that synchronizing follicular wave emergence with DFR and hCG in short synchronization protocols used before superstimulation can increase the success rate of OPU/IVEP programs.

## Data Availability

The datasets used and analyzed in the current study are available from the corresponding author on reasonable request.
